# Effects of NF-kB Signaling Inhibitors on Bed Bug Resistance to Orally Provisioned Entomopathogenic Bacteria

**DOI:** 10.3390/insects12040303

**Published:** 2021-03-30

**Authors:** Jose E. Pietri, Rashaun Potts

**Affiliations:** Division of Basic Biomedical Sciences, Sanford School of Medicine, University of South Dakota, Vermillion, SD 57069, USA; rashaun.potts@usd.edu

**Keywords:** bed bug, cimex, immunity, NF-kB, bacteria, entomopathogen, biological control

## Abstract

**Simple Summary:**

Bed bugs are prolific insect pests across the globe. Chemical control methods are failing at an increasing rate, creating a need for alternative methods. While the use of microbial agents for biological control of insects has had significant success against many pests and holds great promise, research in this area has only recently begun to be considered for bed bugs. Here, we show that chemical inhibition of pathways involved in bed bug immunity can boost mortality during infection with orally provisioned entomopathogenic bacteria by reducing the ability of bed bugs to clear bacterial infection. Our findings suggest that reducing resistance to infection through inhibition of immune signaling may be a viable strategy for biological control of bed bugs. From an applied perspective, this work is a critical step towards the optimization of ingestible biological control formulations.

**Abstract:**

Bed bugs are globally important pests and there is an ongoing need for the development and improvement of bed bug control tools. Though promising against other insect pests, the exploration of biological methods for bed bug control is limited. Previously, we identified several species of bacteria that have entomopathogenic effects against bed bugs when ingested. We also described the conservation of several antibacterial responses in bed bugs, including the expression of immune effector genes regulated by NF-kB transcription factors through the Toll and immune deficiency (IMD) signaling pathways. Accordingly, we predicted that chemical inhibition of NF-kB signaling could reduce bed bug resistance to orally provisioned entomopathogenic bacteria, potentially improving their effectiveness as biological control agents. In the present study, we administered four small molecule inhibitors of NF-kB signaling (BMS345541, IKK16, IMD0354, Takinib) to bed bugs by feeding them in a blood meal. We then quantified basal mortality and mortality in response to oral infection with two different entomopathogenic bacteria (*Pseudomonas entomophila* and *Bacillus thuringiensis israelensis*). None of the NF-kB signaling inhibitors tested increased mortality above control levels when administered alone, suggesting a lack of direct toxicity. However, one inhibitor (IKK16) significantly enhanced the rate of mortality from oral infection with *P. entomophila*. Enhanced mortality was independent of direct effects of IKK16 on *P. entomophila* growth in vitro but was associated with higher bacterial loads in vivo (i.e., reduced resistance). Together, these results provide new insight into the regulation of the bed bug immune system and suggest that administration of entomopathogens in combination with inhibition of immune signaling pathways to reduce infection resistance may be effective for biological control of bed bugs.

## 1. Introduction

Bed bugs, including the common bed bug, *Cimex lectularius*, are insect ectoparasites that live in close association with and feed on the blood of human hosts [1]. Over the last several decades, they have experienced a substantial global resurgence, making them common insect pests in urban environments worldwide [2]. Bed bugs are not only a nuisance, but their bites can also lead to a number of health problems, including: anemia, anxiety, insomnia, allergic reactions, and secondary infections [3]. Countless dollars are spent each year on treatment of infestations, yet there remains significant room for improvement and development of bed bug control tools. Most notably, there are growing concerns regarding the spread of resistance to common chemical insecticides across bed bug populations [4], and other effective non-chemical methods, such as whole-room heat treatments, can be tedious and expensive, limiting their broad application.

The use of microbial agents for biological control of insects is an alternative approach to chemical insecticides that has had significant success against agricultural pests and is being developed for use against medical and structural pests [5,6]. Bed bugs are susceptible to infection with the entomopathogenic fungus *Bauvaria bassiana* and a formulation using this agent (Aprehend) is commercially available [7,8,9]. Besides this product, no other efficacious biological control formulations for bed bugs have been developed. However, our group previously performed a screen of known entomopathogenic bacteria and identified several species with activity against the common bed bug when administered orally, including *Bacillus thuringiensis israelensis* and *Pseudomonas fluorescens* [10]. These are candidate biological control agents. It is also known that because bed bugs mate by traumatic insemination they can naturally become infected with pathogenic bacteria present in the environment during copulatory wounding, leading to post-mating mortality [11,12]. This process could potentially also be exploited for biological control. 

A deeper understanding of bacterial pathogenicity and anti-bacterial immunity in bed bugs is critical in order to most effectively leverage entomopathogenic bacteria to control them. Bed bugs mount diverse immune responses to bacteria, both cellular and humoral. These include phagocytosis, lysozyme-mediated killing, and expression of antimicrobial peptides [13,14,15,16]. If such responses occur following oral administration of biological control agents, as can be reasonably expected, then resistance to infection may be increased and the efficacy of the agents may be reduced. 

**Figure 1:**
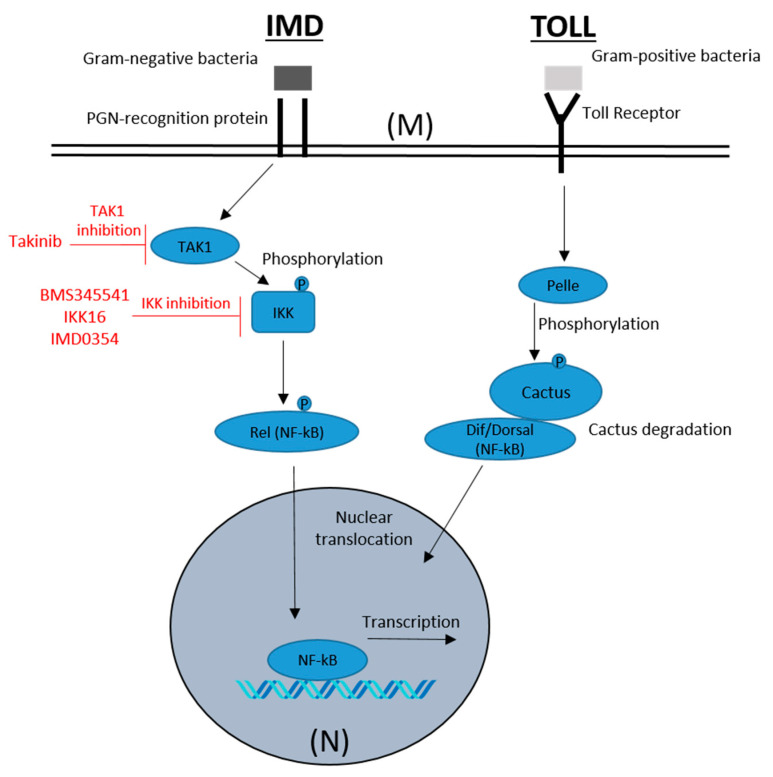
General overview of NF-kB signaling in insects. The immune deficiency (IMD) and Toll pathways are two distinct signaling cascades with similar organization that are activated at the cell membrane (**M**) by sensing of Gram-negative and Gram-positive bacteria, respectively. Activation ultimately leads to the translocation of NF-kB transcription factors (e.g., Relish, Dif/Dorsal) to the cell nucleus (**N**). Nuclear translocation of NF-kB results in transcription of genes involved in immunity, such as antimicrobial peptides. For details, see [17,18,19,20,21,22].

The Toll and immune deficiency (IMD) signaling pathways are the major regulators of humoral anti-bacterial immunity in insects (Figure 1). These pathways, which have been extensively characterized in model insects [17,18,19,20,21,22], transduce receptor-mediated recognition of pathogen associated molecular patterns into the nuclear translocation of NF-kB transcription factors, ultimately inducing the expression of a multitude of effector genes (e.g., antimicrobial peptides). Bed bugs encode and express most components of the Toll and IMD pathways, including kinases (e.g., IKK, TAK1, Pelle), NF-kB transcription factor homologs (i.e., Relish, Dif/Dorsal) and antimicrobial peptides (e.g., Defensin) [15,23], but little is known about their function and regulation, as the anti-microbial repertoire of bed bugs is only beginning to be appreciated.

Studies in the related Hemipteran bug *Rhodnius prolixus* revealed that chemical inhibition of NF-kB signaling using an IKK inhibitor (IMD0354) suppressed expression of antimicrobial peptides and increased mortality during both bacterial and parasitic infections [24]. Based on these findings, we hypothesized that similar effects of NF-kB inhibition are conserved in bed bugs and that these effects could be useful for improving the efficacy of entomopathogens as biological control agents against them. The goal of the present study was to determine if inhibition of NF-kB signaling could reduce resistance to orally administered entomopathogenic bacteria. Accordingly, we fed bed bugs four individual small molecule inhibitors of NF-kB signaling (BMS345541, IKK16, IMD0354, Takinib) alone or in combination with two different bacterial entomopathogens (*Pseudomonas entomophila* and *Bacillus thuringiensis israelensis)* and we examined the effects on both bed bug mortality and bacterial growth.

## 2. Materials and Methods

### 2.1. Bed Bug Rearing

The Cincinnati SRL strain of *Cimex lectularius* was used in the present study. This strain is derived from a population of individuals collected by technicians from Sierra Research Laboratories, Inc. (Modesto, CA, USA) in 2007 and has been maintained under laboratory conditions since this time. Colonies were maintained at the University of South Dakota in plastic jars containing corrugated cardboard harborages at 28 ± 1 °C and 60–70% relative humidity on a 12:12 photoperiod. The colonies were fed aseptically collected defibrinated rabbit blood (Hemostat Laboratories, Dixon, CA, USA) once per week using an artificial membrane system (Hemotek Ltd., Blackburn, United Kingdom).

### 2.2. NF-kB Signaling Inhibitors

**Figure 2:**
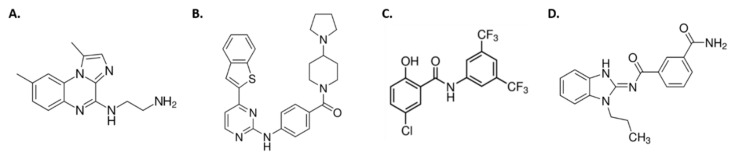
Chemical structures of NF-kB signaling inhibitors. (**A**) BMS345541. (**B**) IKK16. (**C**) IMD0354. (**D**) Takinib.

All chemical inhibitors were purchased from Sigma Aldrich (St. Louis, MO, USA). BMS345541, IKK16, and IMD0354 are specific inhibitors of IkB kinase (IKK) while Takinib is a specific inhibitor of TAK1 (Figure 1 and Figure 2) [25,26,27,28,29]. Working stocks of 5 mg/mL were made in dimethyl sulfoxide (DMSO) for all inhibitors except for Takinib, which was dissolved at 2 mg/mL due to its lower solubility.

### 2.3. Inhibitor Feeding Experiments

Based on concentrations used in other insects [24], inhibitors were further diluted to 5 μg/mL or 10 μg/mL in defibrinated rabbit blood for feeding to bed bugs to examine their effects as lone agents. Bed bugs fed equivalent volumes of DMSO diluent in blood served as controls in these experiments. In brief, groups consisting of roughly even numbers of adult male and female bed bugs that had not blood fed for at least seven days were provided access to blood treated with inhibitors using the artificial membrane system (Hemotek Ltd.) until fully engorged. Mortality was then monitored regularly over a period of seven days. Two independent biological replicates consisting of 10–15 insects per group were carried out and cumulative survival at the end of the seven-day period was compared using chi-square testing. 

### 2.4. Bacterial Entomopathogen Cultures

Two known bacterial entomopathogens were used in the present study. The Gram-positive bacterium *Bacillus thuringiensis israelensis* was obtained from Carolina Biological Supply (Burlington, NC, USA). This bacterium was investigated because our previous studies demonstrated it has activity against bed bugs when ingested [10]. The Gram-negative bacterium *Pseudomonas entomophila* was obtained from the German Collection of Microorganisms and Cell Cultures (DSMZ, Braunschweig, Germany). This bacterium is a relative of *Pseudomonas fluorescens*, which we previously showed has activity against bed bugs when ingested [10] but is known to have stronger entomopathogenic effects in other species [30,31]. Stocks of these bacteria were grown overnight in Luria-Bertani (LB) medium on a shaker at room temperature.

### 2.5. Effects of NF-kb Signaling Inhibitors on Bacterial Entomopathogen Cultures

To determine if inhibitors had any direct impact on the growth of entomopathogenic bacteria prior to their administration in vivo, cultures of each bacterium were inoculated into LB medium and individual inhibitors were added to the cultures at a final concentration of 10 μg/mL. Cultures containing equal volumes of DMSO diluent served as controls. After shaking overnight at room temperature, growth of bacteria in the presence of inhibitors was quantified by measuring optical density of the cultures at 600 nm (OD_600_) on a spectrophotomer. OD_600_ values of cultures containing inhibitors were then normalized to DMSO controls. Three replicates were conducted with each inhibitor and growth was compared to controls (set at 1) using a 1-sample *t*-test.

### 2.6. Combination Treatments with NF-kB Signaling Inhibitors and Bacterial Entomopathogens

To determine if NF-kB signaling inhibitors could enhance mortality from orally provisioned entomomopathogenic bacteria, cultures of each bacterium were grown overnight by shaking at room temperature. The following day, cultures of *P. entomophila* were standardized to an OD_600_ value of 2.3 (3 × 10^8^ colony forming units/mL) and cultures of *B. thuringiensis isralensis* were standardized to an OD_600_ value of 2.2 (7 × 10^7^ colony forming units/mL), based on their overnight growth across replicates. *P. entomophila* was then further diluted 1:100 in defibrinated rabbit blood, whereas *B. thuringiensis israelensis* was further diluted 1:50 in defibrinated rabbit blood due to its generally lower virulence [10]. Inhibitors or DMSO diluent were added to the blood meal containing diluted bacteria to a final concentration of 10 μg/mL. Groups consisting of roughly even numbers of adult male and female bed bugs that had not blood fed for at least seven days were provided access to treated blood meals using the artificial membrane system (Hemotek Ltd.) until fully engorged. Short-term mortality was then monitored over a period of three days, consistent with our previous studies of entomopathogenic bacteria in bed bugs [10]. Groups of bed bugs fed DMSO diluent in blood were included as controls for infection experiments. Three independent biological replicates with 10–14 insects per group were carried out and cumulative survival curves were compared using the Gehan-Breslow-Wilcoxon test.

### 2.7. Estimation of Bacterial Entomopathogen Load In Vivo

To determine if enhanced mortality during *P. entomophila* infection was associated with increased bacterial load (reduced resistance), adult male bed bugs that had not fed for at least seven days were provided access to blood meals containing *P. entomophila* with or without 10 ug/mL of IKK16 using the artificial membrane system (Hemotek Ltd.) until fully engorged. The two treatments were prepared as described in the preceding section of the methods (combination treatments). Then, 24 h later, live and dead bed bugs from each infected treatment group were collected. Individual insects were surface sterilized by rinsing with 10% bleach and 70% ethanol and homogenized in sterile phosphate buffered saline (PBS). Homogenates were then serial diluted in PBS and plated on LB agar plates to estimate the load of *P. entomophila* bacteria expressed as colony forming units (CFU) per insect after 24 h of incubation at 28 C. As culturable bed bug commensals in the gut are minimal [32] and slow growing, this methodology allowed us to specifically quantify *P. entomophila.* Two independent biological replicates of the experiment were conducted (15 individual insects examined per treatment group in total). The insects in each treatment were further subdivided into dead and live categories for analysis. One outlier value was removed from the live *P. entomophila* treated group based on the results of ROUT testing. *P. entomophila* loads were compared using ANOVA with adjustment for multiple comparisons.

## 3. Results

### 3.1. Toxicity of NF-KB Signaling Inhibitors as Lone Agents

**Figure 3:**
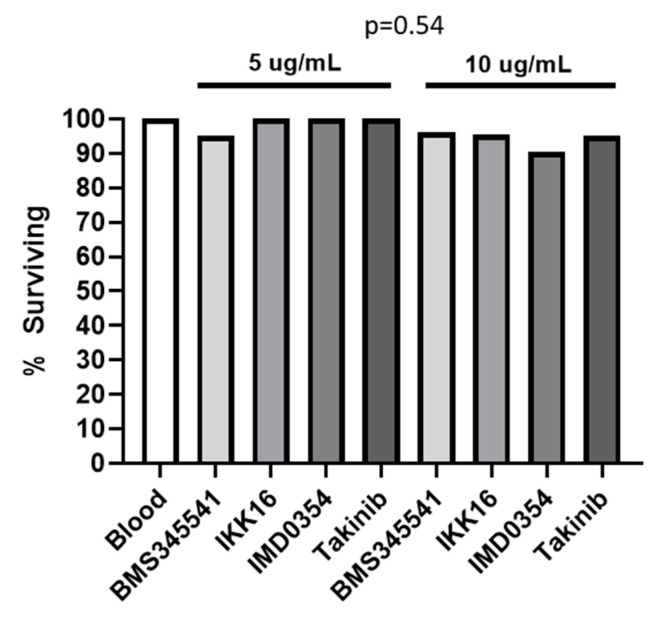
Seven-day survivorship of bed bugs fed NF-kB signaling inhibitors. Inhibitors were dissolved in DMSO and diluted in a blood meal to a final concentration of 5 μg/mL or 10 μg/mL. Shown is cumulative survival from 2 independent biological replicates of 10–15 adult insects per treatment group assessed 7 days post-feeding. Differences between treatment groups were not statistically significant as determined by Chi-square testing.

None of the NF-kB signaling inhibitors tested (BMS345541, IKK16, IMD0354, Takinib), whether provisioned at 5 μg/mL or 10 μg/mL, elicited significant mortality relative to DMSO controls (Figure 3, chi-square test, *p =* 0.54). In fact, survival at 7 days post-feeding remained above 90% in all control groups and in all groups treated with inhibitors. As such, only two independent replicates of these experiments were performed due to a lack of observed effect that was consistent in both replicates. These results indicated that NF-kB inhibitors are not directly toxic to bed bugs at the doses given.

### 3.2. Effects of NF-kB Signaling Inhibitors on Bacterial Entomopathogen Growth In Vitro

**Figure 4:**
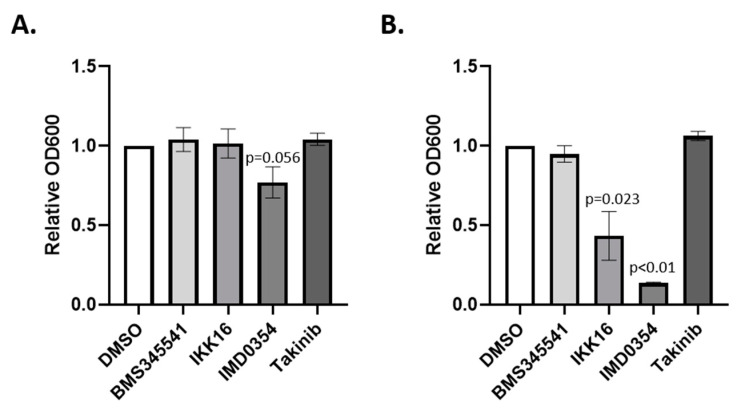
Direct effects of NF-kB signaling inhibitors on entomopathogenic bacteria in culture. Inhibitors dissolved in DMSO were added to bacterial cultures at a final concentration of 10 μg/mL. Cultures were shaken overnight at room temperature and bacterial growth was quantified by measurement of OD_600_ on a spectrophotometer. (**A**) *Pseudomonas entomophila.* (**B**) *Bacillus thuringiensis israelensis*. Shown is the mean ± SEM from 3 independent biological replicates. Differences in growth relative to the DMSO control (set at 1) were assessed by 1 sample *t*-test.

Prior to examining the effects of NF-kB signaling inhibitors on resistance to infection with entomopathogenic bacteria in vivo, we sought to determine if these compounds had any direct effects on entomopathogenic bacteria of interest, which could have confounded the interpretation of in vivo experiments. To do so, we cultured *B. thuringiensis* and *P. entomophila* in LB medium containing 10 μg/mL of each inhibitor in vitro (Figure 4). In these experiments, IMD0354 inhibited the growth of both *P. entomophila* (*t*-test, *p =* 0.056) and *B. thuringiensis* (*t*-test, *p* < 0.01). Similarly, IKK16 inhibited the growth of *B. thuringiensis* (*t*-test, *p =* 0.024). Therefore, these combinations were excluded from further study. On the other hand, BMS345541, IKK16, and Takinib did not affect the growth of *P. entomophila*, and BMS345541 and Takinib did not affect the growth of *B. thuringiensis.* These combinations were selected for further study.

### 3.3. Effects of NF-kB Signaling Inhibitors on Mortality during Infection with Entomopathogenic Bacteria

**Figure 5:**
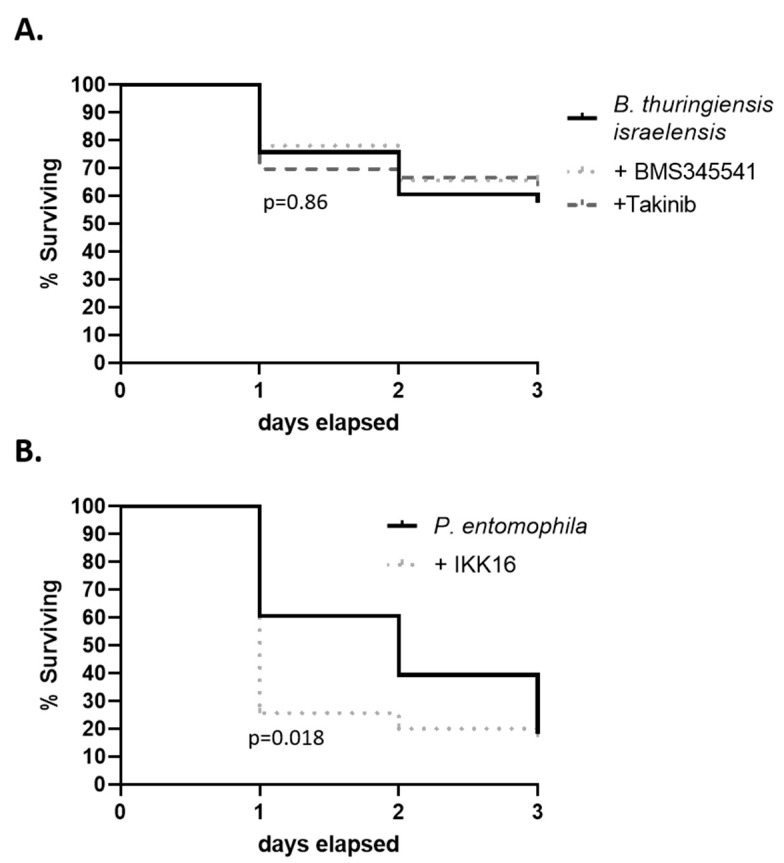
Three-day survivorship of bed bugs fed combinations of NF-KB signaling inhibitors and entomopathogenic bacteria. Inhibitors at a final concentration of 10 μg/mL were combined in a blood meal with (**A**) *Bacillus thuringiensis israelensis* or (**B**) *Pseudomonas entomophila.* Shown is the cumulative survival from 3 independent biological replicates of 10–14 adult insects per group. All three replicates produced consistent patterns and no mortality was observed in blood controls. Survival curves were compared using the Gehan–Breslow–Wilcoxon test.

When NF-kB signaling inhibitors were administered together with entomopathogenic bacteria, variable effects were observed. In these experiments, 10 μg/mL of BMS345541 or Takinib had no effect on mortality during infection with *B. thuringiensis* (Figure 5A, Gehan-Breslow test, *p =* 0.86). The same inhibitors also had no effect on mortality during *P. entomophila* infection (not shown). However, when *P. entomophila* was combined with 10 μg/mL of IKK16, the rate of death was significantly increased relative to insects infected with the bacterium alone (Figure 5B, Gehan-Breslow test, *p =* 0.018). Mortality at day 1 was 39.4% in the *P. entomophila* treated group but was increased to 74.3% when combined with IKK16. Similarly, at day 2, mortality in the *P. entomophila* group was 60.6% but was increased to 80% when combined with IKK16. At day 3, mortality was 81.8% in the *P. entomophila* group and 82.9% when combined with IKK16. In three replicates of these infection experiments, mortality patterns were consistent, and no mortality was observed in control groups fed DMSO diluent in blood (not plotted). The results demonstrated that during infection some NF-kB signaling inhibitors can act as synergists of bacterial entomopathogens to enhance the rate of mortality, which in an important parameter in the context of control, but these effects are specific to only certain combinations.

### 3.4. Pseudomonas Entomophila Load during Infection of Bed Bugs

**Figure 6:**
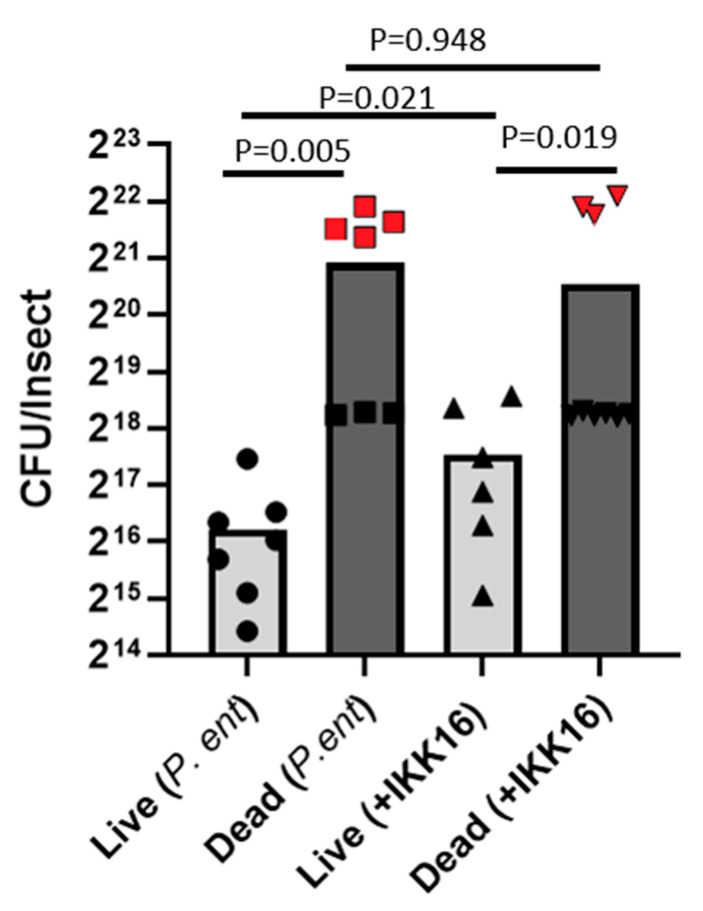
*Pseudomonas entomophila* load during in vivo infection. Adult bed bugs were fed a blood meal containing *P. entomophila* with or without 10 ug/mL of IKK16 added. At 24 h post-feeding, living and dead insects were surface sterilized, homogenized and plated on LB agar to estimate the load of *P. entomophila* (expressed as CFU/insect). Shown are individual data points derived from 2 independent biological replicates and their mean. Bacterial loads were compared using ANOVA with adjustment for multiple comparisons. Red data points indicate insects suspected of having experienced post-mortem bacterial replication which were excluded from the statistical analysis.

The bacterial load upon death (BLUD) is an intrinsic property of bacterial pathogens [33]. That is, different pathogens consistently kill their hosts once a threshold bacterial load is reached. In general, the survival of an organism during a pathogenic infection can be altered in one of two ways. The organism can eliminate the pathogen, reducing its load, which is known as resistance, or it can modulate aspects of its own physiology to reduce damage from the infection without reducing pathogen load, which is known as tolerance [34]. Inhibition of NF-kB mediated immune responses is a mechanism that reduces resistance, since the effectors regulated by this pathway are well known to directly eliminate microbes [19]. To determine if mortality during the combination treatment of *P. entomophila* and IKK16 was associated with the expected mechanism of reduced resistance (higher bacterial loads) rather than reduced tolerance (death at lower bacterial loads), bacterial loads in insects fed *P. entomophila* alone or in combination with IKK16 were compared 24 h post-infection (Figure 6).

By 24 h post-infection, significantly lower survival was observed in the infected group treated with IKK16 (Figure 5B). Bed bugs that died from infection by 24 h post-feeding exhibited a bimodal distribution of bacterial loads [35]. One cluster of dead bed bugs harbored lower CFU levels comparable to the upper threshold seen in live insects, suggesting these insects had only recently died and were near the BLUD (Figure 6). Meanwhile, another cluster of dead bed bugs harbored exponentially more *P. entomophila* CFUs. In these insects, some post-mortem bacterial replication may have occurred [36] and they were removed from statistical analysis to make a more conservative comparison between live and dead individuals. Whether fed *P. entomophila* alone (ANOVA, *p =* 0.005) or in combination with IKK16 (ANOVA, *p =* 0.091), dead bed bugs harbored higher *P. entomophila* loads than those that remained alive. Further, bed bugs treated with IKK16 that remained alive 24 h-post infection harbored higher loads of *P. entomophila* than live counterparts not treated with the inhibitor (ANOVA, *p =* 0.021). Together, these data suggest that IKK16 boosts mortality by reducing the innate ability of bed bugs to clear the pathogen (i.e., reduces resistance), since the inhibitor did not have any effects on bed bugs when given alone (Figure 3), did not affect *P. entomophila* growth in vitro (Figure 4), and did not affect tolerance (produce mortality with lower bacterial loads) in vivo (Figure 6).

## 4. Discussion

Here, we demonstrate that provisioning of a small molecule inhibitor of NF-kB signaling (IKK16) can increase the rate of bed bug mortality during infection with a bacterial entomopathogen (*P. entomophila*). We further show that increased mortality is independent of direct effects of the inhibitor on bacterial growth and is instead the result of a reduced ability of bed bugs to clear the infection (i.e., reduced resistance) [34] when treated with the inhibitor. These findings provide new information regarding the regulation of bed bug immunity and indicate that reducing bed bug resistance to infection through inhibition of innate immune signaling may be a viable strategy for biological control. In addition, ours is the first report that *P. entomophila* is pathogenic to bed bugs, expanding the known range of hosts this well-characterized entomopathogen is active against [30,31].

Although administration of 10 ug/mL of the inhibitor IMD0354 to the triatomine *R. prolixus* elicited significant mortality alone in other studies [24], we did not observe the same phenomenon in *C. lectularius* with IMD0354 or any other inhibitor given at the same dose. This difference is indicative of differential susceptibility to the direct toxicity of this class of compounds across hemipterans and suggests that some similar compounds may be useful chemical control agents for insects regardless of their effects on resistance to entomopathogens.

In selecting combinations of entomopathogens and immune signaling inhibitors for further study and development, it is critical to consider the interaction between these two components independent of the bed bug host. For example, we show that IMD0354 has negative effects on the growth of both *P. entomophila* and *B. thuringiensis* in vitro. Since bacterial proliferation appears to be a key component of pathogenicity for *P. entomophila* and presumably many other entomopathogens [33], such interactions could result in antagonistic effects in vivo in some cases. Indeed, when we preliminarily tested the combination of *P. entomophila* with IMD0354, mortality was lower than in insects fed the bacteria alone (not shown).

It is also important to consider the specificity of synergy between entomopathogens and immune signaling inhibitors. In our studies, several combinations had no effect on mortality relative to bacteria administered alone. The reasons for this specificity remain undetermined, but we speculate a number of factors are at play. The differential importance of Toll and IMD mediated immune responses in controlling Gram-positive vs. Gram-negative bacteria likely plays a role. *B. thuringiensis*, which was not affected by inhibitors of TAK1 or IKK (Takinib, BMS345541), is Gram-positive, while *P. entomophila*, which was enhanced by an inhibitor of IKK (IKK16) is Gram-negative. Immune responses to Gram-positive and Gram-negative bacteria are controlled by distinct signaling proteins and NF-kB homologs in the Toll and IMD pathways, respectively [17,18,19,20,21,22]. In *Drosophila*, IKK is involved in IMD but not Toll signaling [18]. It is therefore somewhat expected that inhibition of IKK in the IMD pathway would result in differential effects on Gram-positive vs. Gram-negative infections, affecting the latter more significantly. A similar phenomenon was observed during treatment of *R. prolixus* with the IKK inhibitor IMD0354, which differentially influenced mortality from *Staphylococcus aureus* and *Escherichia coli* infection [24]. However, it is noteworthy that in those studies, IKK inhibition still affected both Gram-positive and Gram-negative infections to some degree, suggesting that IKK may be involved in both the Toll and IMD pathways to some extent in hemipterans and that these pathways could perhaps be regulated in ways that diverge from *Drosophila*. In addition, inhibitors may have differential abilities to block bed bug homologs of their targets, which may also contribute to varied effects when combined with bacteria. For instance, BMS345541 may not inhibit bed bug IKK with the same affinity as IKK16, while Takinib may not have high affinity for bed bug TAK1, which would result in a lack of effect for these two inhibitors.

The compounds we tested are known to inhibit IKK and TAK1 with high specificity in other organisms. Nonetheless, a limitation of our work is that we could not pinpoint effects on specific kinases to rule out possible off target effects that influence infection resistance and contribute to synergy. Therefore, additional studies to characterize the mechanisms of bed bug Toll and IMD signaling and to confirm the precise mechanisms by which compounds such as IKK16 influence resistance during infection (e.g., reduction in antimicrobial peptide expression) are of fundamental interest. The goal of our initial work was only to examine the feasibility of combining entomopathogenic bacteria and NF-kB signaling inhibitors. Mechanistic molecular work and more extensive toxicological studies to identify other useful pathogen-inhibitor combinations and their maximally effective doses will inform the optimization of ingestible biological control formulations. Together with the development of ingestible baits that enable delivery [37], which is of ongoing interest, the development of these novel biological control formulations holds promise for enhancing integrated management of bed bugs.

## Data Availability

All relevant data are reported in the manuscript.
